# Habitat complexity and lifetime predation risk influence mesopredator survival in a multi-predator system

**DOI:** 10.1038/s41598-020-73318-3

**Published:** 2020-10-20

**Authors:** Laura C. Gigliotti, Rob Slotow, Luke T. B. Hunter, Julien Fattebert, Craig Sholto-Douglas, David S. Jachowski

**Affiliations:** 1 Department of Forestry and Environmental Conservation, Clemson University, 261 Lehotsky Hall, Clemson, SC USA; 2 Centre for Functional Biodiversity, School of Life Sciences, University of KwaZulu-Natal, Pietermaritzburg, South Africa; 3 Wildlife Conservation Society, Bronx, NY USA; 4 Wyoming Cooperative Fish and Wildlife Research Unit, Department of Zoology and Physiology, University of Wyoming, Laramie, WY USA; 5 andBeyond Phinda Private Game Reserve, Hluhluwe, South Africa; 6 Present Address: Department of Environmental Science, Policy, and Management, University of California Berkeley, 3 Mulford Hall, Berkeley, CA 94720 USA

**Keywords:** Community ecology, Population dynamics

## Abstract

Variability in habitat selection can lead to differences in fitness; however limited research exists on how habitat selection of mid-ranking predators can influence population-level processes in multi-predator systems. For mid-ranking, or mesopredators, differences in habitat use might have strong demographic effects because mesopredators need to simultaneously avoid apex predators and acquire prey. We studied spatially-explicit survival of cheetahs (*Acinonyx jubatus*) in the Mun-Ya-Wana Conservancy, South Africa, to test hypotheses related to spatial influences of predation risk, prey availability, and vegetation complexity, on mesopredator survival. For each monitored cheetah, we estimated lion encounter risk, prey density, and vegetation complexity within their home range, on short-term (seasonal) and long-term (lifetime) scales and estimated survival based on these covariates. Survival was lowest for adult cheetahs and cubs in areas with high vegetation complexity on both seasonal and lifetime scales. Additionally, cub survival was negatively related to the long-term risk of encountering a lion. We suggest that complex habitats are only beneficial to mesopredators when they are able to effectively find and hunt prey, and show that spatial drivers of survival for mesopredators can vary temporally. Collectively, our research illustrates that individual variation in mesopredator habitat use can scale-up and have population-level effects.

## Introduction

Understanding how individual habitat use can influence fitness can offer insight into the structure of food webs and predator–prey interactions^1^. Spatial and temporal variation in resources, as well as the ability of individuals to find and use these resources, can lead to differences in individual survival and reproduction, which in turn can scale up to population-level effects^2,3^. In predator–prey systems, environmental features that influence the predator’s ability to find or kill prey can affect the survival or reproduction of the predator^4,5^. On the other hand, environmental features that influence predator avoidance, predator detection, the prey’s ability to escape predators, or the prey’s ability to find food, can affect the survival or reproduction of the prey^6–8^. Identifying the connections between habitat use and demography is important for understanding the fitness costs and benefits of habitats^6^, although this understanding is lacking for systems with multiple predators.

In systems with multiple predators, the interaction between habitat use and demography could be particularly complex. Subordinate predators, or mesopredators^9^, need to select habitats that will allow them to obtain prey, while still avoiding predation by top predators^9^. In some systems, apex predators and mesopredators occur in the same general habitats, but mesopredators use fine-scale spatial or temporal partitioning to reduce encounter rates^10,11^. Although this partitioning might reduce short-term mortality risk for mesopredators, the longer-term risk of co-occurring with apex predators could reduce the fitness of the mesopredator through non-consumptive effects, such as reduced foraging opportunities or shifts into non-optimal habitats^12^. Many previous studies have investigated how apex predators can affect the abundances^13–15^, habitat use^16,17^, and behavior of mesopredators^18,19^, but considerably less research has focused on how mesopredator-apex predator cooccurrence can scale-up and affect mesopredator demographic rates, especially across longer-term time scales.

Additionally, habitat characteristics such as vegetation complexity can modulate the habitat-survival relationship in systems with multiple predators. Theory predicts that mesopredators will experience reduced mortality from apex predators (i.e., intraguild predation or intraspecific killing) in areas with high habitat complexity, because of lower encounter rates^20^. Most previous research on the role of habitat complexity on survival has been conducted in experimental systems with aquatic or insect species^20,21^, whereas less research has focused on wild mesopredator populations. The effects of vegetation complexity on mesopredator in wild population might be particularly complicated because of tradeoffs between hunting and protection from apex predators in different habitat types^1^. A greater understanding of how habitat characteristics such as vegetation complexity can influence mesopredator survival, is critical.

We studied the interaction between habitat use and mesopredator demography, using the cheetah (*Acinonyx jubatus*) as our focal species. Cheetahs are subordinate to lions, and the majority of cheetah mortality is from lion predation^22^. In addition to direct predation, lions can steal prey from cheetahs^23^, and can affect the habitat use and behavior of cheetahs^24,25^. In turn, these non-consumptive effects related to predation risk might affect the long-term survival and fitness of cheetahs. Previous research suggests that cheetahs and lions exhibit high levels of home range overlap, but that cheetah use fine-scale spatial partitioning to avoid the short-term risk of lion predation^10,26,27^. However, how this partitioning might indirectly affect the survival of cheetahs in the long-term is unknown. In addition, dense vegetation is hypothesized as another mechanism of lion-cheetah coexistence by acting as a predation refuge^28^, but limited research has focused on the linkages between vegetation and cheetah survival, particularly in the southern portion of their range^29^.

We investigated support for three competing hypotheses of spatial drivers on mesopredator survival: (1) mesopredator survival would be driven by the risk of encountering top predators (top-down spatial regulation), (2) mesopredator survival would be driven by prey densities (bottom-up spatial regulation), and (3) mesopredator survival would be driven by vegetation complexity (habitat complexity risk mediation hypothesis). Under the spatial top-down hypothesis we predicted that cheetah survival would be negatively related to the probability of encountering lions. Under the spatial bottom-up hypothesis we predicted that cheetah survival would be positively related to spatial prey density. Under the habitat complexity risk mediation hypothesis, we predicted that cheetah survival would be positively related to vegetation complexity. Because the strength of spatial drivers might depend on the temporal scale at which they are assessed, we tested our three hypotheses in relation to both short-term and long-term habitat use. By studying spatial influences of mesopredator demography, we can better understand factors that structure food webs with multiple predators, and in turn prioritize habitat features that promote the coexistence of multiple predator species.

## Results

### Short-term spatial drivers of survival

We included 133 cheetahs in our survival analyses for a total of 110 months. Within these cheetahs, 28 individuals were only in the adult state, 78 individuals were only in the cub state, and 27 individuals were included in the models as both in the cub and adult states.

Cheetah survival was most sensitive to short-term environmental conditions within the 50% home range contour and the same top model was supported regardless of home range contour (Appendix S2). Thus, we only present the results from the 50% HR models. At the short-term time scale, cheetahs exhibited variation in environmental conditions within their home range with regards to EVI (mean = 0.29; range = 0.14–0.44), lion encounter risk (mean = 0.36; range = 0.16–0.90), and prey density (mean = 37.8 prey/km^2^, range = 24.0–80.3 prey/km^2^).

**Figure 1:**
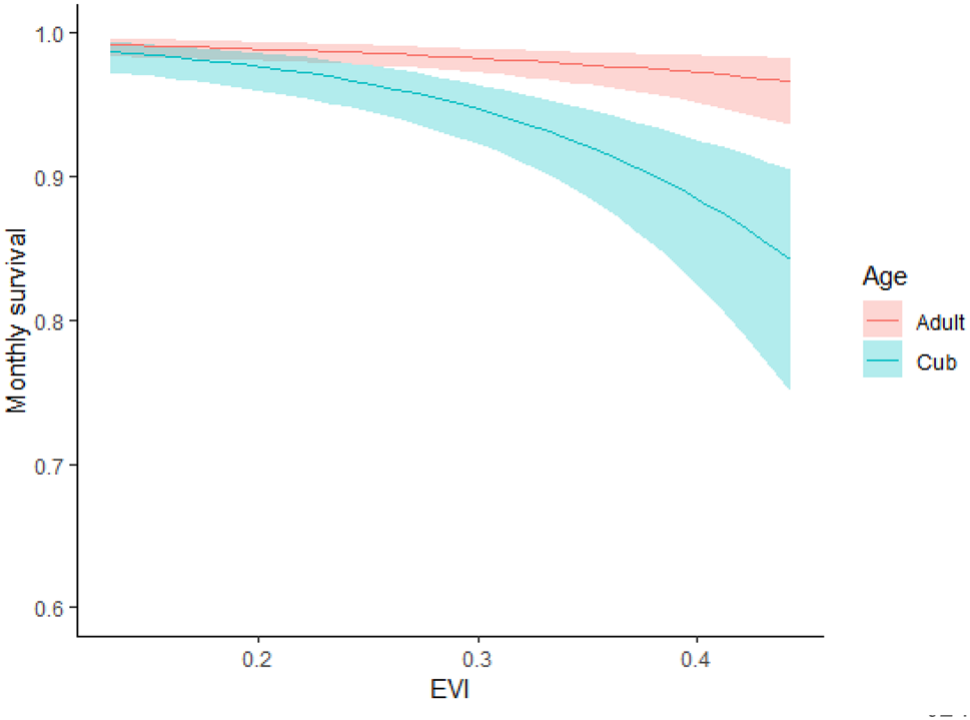
Monthly survival of adult and cheetah cubs in relation to short term (seasonal) average Enhanced Vegetation Index (EVI) within an individual’s home range, Mun-Ya-Wana Conservancy, KwaZulu-Natal, South Africa, 2008–2018. Shaded regions represent 85% CI.

For our short-term survival models, survival was best described by the average EVI within the core of a cheetah’s home range (Table 1). In contrast to our predictions under the habitat complexity risk mediation hypothesis, for both adults and cubs, EVI had a negative influence on survival, with higher survival occurring at the lowest EVI values (Fig. 1). At the lowest EVI values, adult monthly survival was 0.99 (85% CI = 0.98–1.00) and cub monthly survival was 0.98 (85% CI = 0.97–0.99), whereas at the highest EVI values, adult monthly survival was 0.97 (85% CI = 0.94–0.98) and cub monthly survival was 0.84 (85% CI = 0.75–0.91). Cub survival was more sensitive than adult survival to changes in EVI, with the probability of surviving decreasing 2.8% for every 1-unit increase in EVI.

**Table 1:** Model selection results for multi-state joint live-encounter dead-recovery spatial-explicit survival models for cheetahs with seasonal spatial covariates, Mun-Ya-Wana Conservancy, KwaZulu-Natal, South Africa, 2008–2018.

Model	AICc	ΔAIC_C_	− 2 × ln(*L*)^a^	w^b^	k^c^
S(state:EVI)	3601.15	0	3583.02	0.55	9
S(state:lion + state:EVI)	3603.51	2.36	3581.32	0.17	11
S(state:lion * state:EVI)	3604.06	2.91	3577.80	0.13	13
S(state:prey + state:EVI)	3604.60	3.45	3582.41	0.10	11
S(state:EVI + state:lion + state:prey)	3607.08	5.93	3580.82	0.03	13
S(state:prey * state:EVI)	3607.31	6.16	3581.05	0.03	13
S(state:lion * state:prey)	3613.35	12.20	3587.09	0.00	13
S(state:prey)	3617.96	16.81	3599.84	0.00	9
S(state)	3619.33	18.19	3603.23	0.00	8
S(state:lion)	3620.62	19.47	3602.49	0.00	9
S(state:lion + state:prey)	3621.51	20.36	3599.32	0.00	11

States in the model include cubs (juveniles dependent on their mothers) and adults (non-juveniles). All models include effects of year on recovery rates and season on survival rates.

^a^Log-likelihood.

^b^Akaike model weight.

^c^Number of model parameters.

### Long-term spatial drivers of survival

Similar to short-term survival, we assessed lifetime environmental covariates using the 50% HR contour. At the lifetime scale, cheetahs exhibited variation in environmental conditions within their home range with regards to EVI (mean = 0.3; range = 0.19–0.42), lion encounter risk (mean = 0.37; range = 0.13–0.84), and prey density (mean = 40.4 prey/km^2^, range = 25.8–75.7 prey/km^2^).

**Figure 2:**
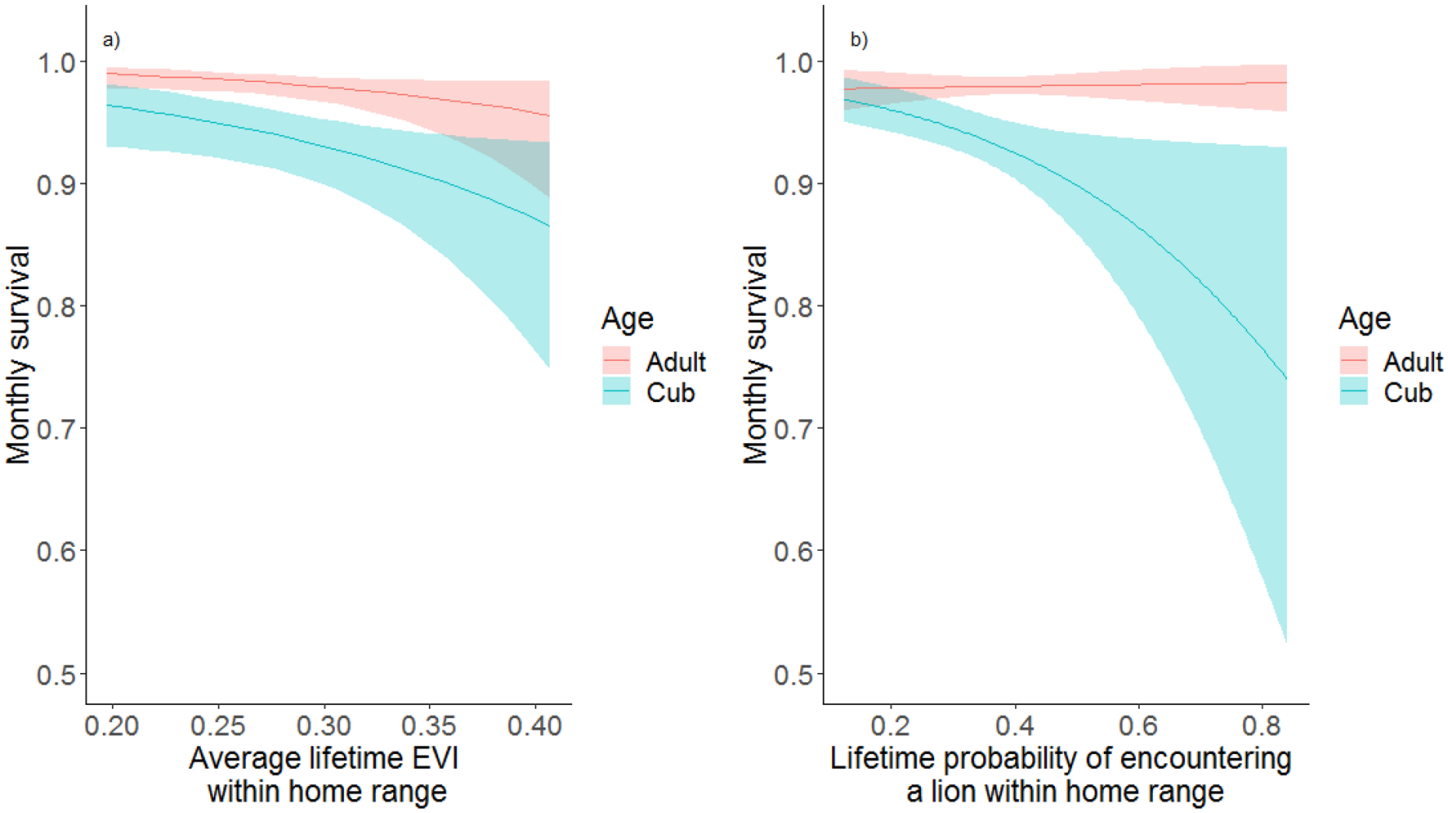
Monthly survival of adult and cheetah cubs in relation to (**a**) long-term (lifetime) average Enhanced Vegetation Index (EVI) within an individual’s home range while holding lion density constant at an average value, and (**b**) long-term (lifetime) average probability of encountering a lion within an individual’s home range while holding EVI constant at an average value, Mun-Ya-Wana Conservancy, KwaZulu-Natal, South Africa, 2008–2018. Shaded regions represent 85% CI.

When considering environmental covariates across individuals’ lifetimes, survival was best described by a model including the average EVI and the average risk of encountering a lion within a cheetahs’ home range during their lifetime (Table 2). Models including EVI and lion encounter risk separately were also competitive (Table 2), however we only present results from the top model given that the covariate relationships were similar in all competitive models. In contrast to our predictions under the habitat complexity risk mediation hypothesis, lifetime EVI had a negative influence on adult and cub survival. At the lowest lifetime EVI values, adult monthly survival was 0.99 (85% CI = 0.98–1.00) and cub monthly survival was 0.96 (85% CI = 0.93–0.98), whereas at the highest lifetime EVI values, adult monthly survival was 0.96 (85% CI = 0.89–0.98) and cub monthly survival was 0.87 (85% CI = 0.75–0.93). As predicted by the spatial top down hypothesis, lifetime lion encounter risk had a negative influence on cub survival, although there was not a significant effect of lifetime lion encounter risk on adult survival (Fig. 2). For cubs, at the lowest lifetime lion encounter risk, monthly survival was 0.97 (85% CI = 0.95–0.99), whereas at the highest lifetime lion encounter risk, monthly survival was 0.74 (85% CI = 0.52–0.96).

**Table 2:** Model selection results for multi-state joint live-encounter dead-recovery spatial-explicit survival models for cheetahs with spatial covariates averaged across individual cheetahs’ lifetimes, Mun-Ya-Wana Conservancy, KwaZulu-Natal, South Africa, 2008–2018.

Model	AICc	ΔAIC_C_	− 2 × ln(*L*)^a^	w^b^	k^c^
S(state:lion + state:EVI)	3608.85	0	3586.66	0.30	11
S(state:EVI)	3609.06	0.21	3590.93	0.27	9
S(state:lion)	3610.45	1.60	3592.32	0.13	9
S(state:prey * state:EVI)	3611.52	2.67	3585.26	0.08	13
S(state:lion * state:EVI)	3611.82	2.97	3585.56	0.07	13
S(state:EVI + state:lion + state:prey)	3612.36	3.51	3586.10	0.05	13
S(state:prey + state:EVI)	3612.44	3.59	3590.25	0.05	11
S(state:lion * state:prey)	3613.40	4.55	3587.14	0.03	13
S(state:lion + state:prey)	3614.41	5.56	3592.22	0.02	11
S(state:prey)	3618.74	9.89	3600.61	0.00	9
S(state)	3619.33	10.49	3603.23	0.00	8

States in the model include cubs (juveniles dependent on their mothers) and adults (non-juveniles). All models include effects of year on recovery rates and season on survival rates.

^a^Log-likelihood.

^b^Akaike model weight.

^c^Number of model parameters.

## Discussion

We evaluated support for effects of spatial influences of top-down predation risk, bottom-up prey availability, and habitat complexity on cheetah survival, and found the most consistent support for survival being influenced by habitat complexity across multiple temporal scales. However, our results contradict the habitat complexity risk mediation hypothesis, which predicts that mesopredators should experience increased survival in areas of high habitat complexity^20^. Instead, our results show that subordinate predators do not always benefit from structurally complex habitats, potentially because subordinate predators might only benefit from habitat complexity if they are able to avoid predation, and effectively obtain prey, in complex habitats. In predator–prey systems, using specific areas as refuges from predation can come at a cost to the prey, because although they might reduce predation risk, the resource availability of the refuge might be lower than non-refuge areas because of increased competition or sub-optimal conditions^30,31^. In systems of multiple predators, the quality of a predation refuge habitat might be related to the subordinate predator’s ability to find and hunt prey, which is a function of the subordinate predator’s hunting mode^32^.

**Figure 3:**
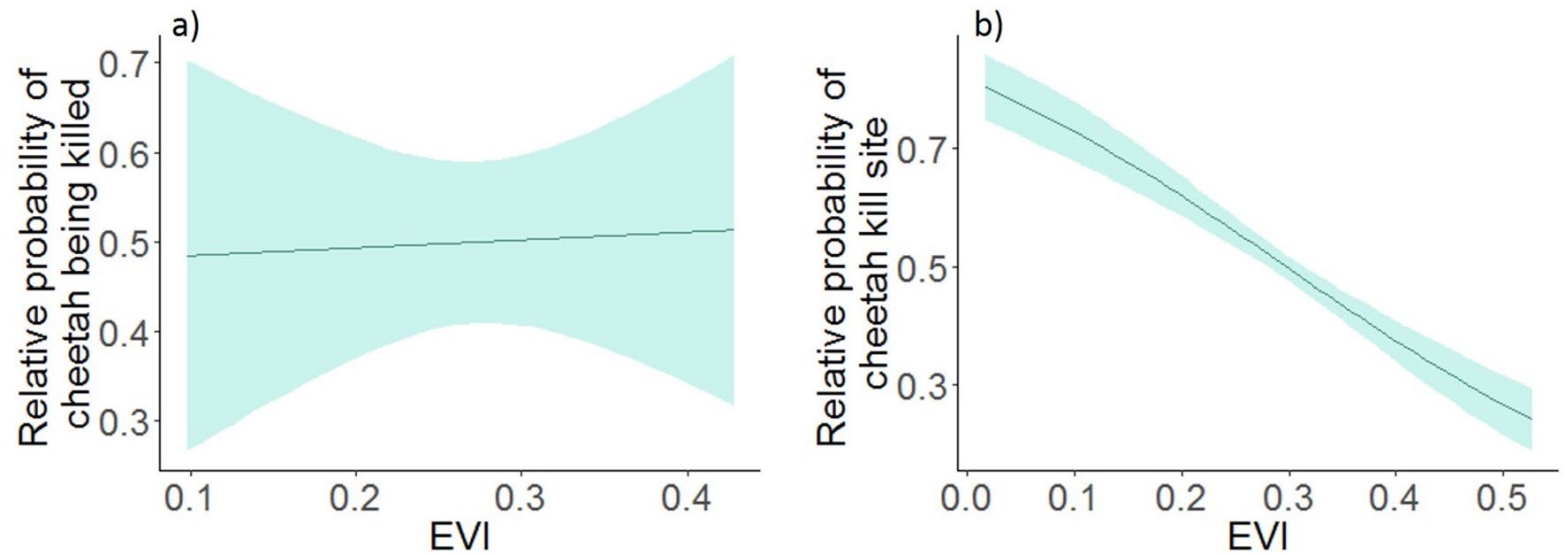
Relative probability of (**a**) cheetahs being killed by other predator species and (**b**) cheetah kill site occurrence in relation to Enhanced Vegetation Index (EVI), Mun-Ya-Wana Conservancy, KwaZulu-Natal, South Africa, 2008–2018. Shaded regions represent 85% CI.

There are two main explanations as to why we did not find support for the habitat complexity risk mediation hypothesis in our study system. First, vegetation complexity might increase or reduce the probability of cheetahs being predated upon. Ambush predators in a variety of systems have been found to have enhanced hunting abilities in structurally-complex areas because of decreased sight lines for prey^33,34^. Although lions use a variety of habitat types, they kill prey more frequently in areas of dense vegetation^35,36^. Closed habitat types could act as a predation refuge for cheetahs by providing cover to enhance concealment from lions, but these habitats could also increase predation risk because of the hunting preferences of lions. Conversely, open habitat types might reduce the probability of predation by improving cheetahs’ ability to detect nearby lions, compared to closed habitats^37^. However, when we analyzed locations where cheetahs were killed by other predators (Appendix S3), we did not find evidence to suggest that vegetation complexity increased the risk of cheetahs being predated upon (Fig. 3a). Therefore, it seems that cheetahs experience predation independent of vegetation complexity, and higher predation in areas of higher EVI might not be the mechanism driving our observed patterns of spatial survival.

Second, vegetation complexity might influence cheetahs’ hunting ability, which in turn could affect survival. Cheetahs are coursing predators, as opposed to ambush predators, and can reach high speeds when chasing prey^38^. Therefore, open areas could improve cheetah hunting success by allowing cheetahs to see prey easier and facilitating high-speed chases. Although cheetahs are able to hunt in areas of dense vegetation^39^, they are more likely to initiate hunts, and have higher hunting success, in open habitats^40^. Prey availability can be an important driver of carnivore demography^41^, so the use of areas to facilitate hunting, rather than the density of prey themselves^42^, could influence mesopredator survival. Indeed, when we analyzed cheetah kill site locations (Appendix S3) we found that cheetah kill sites were more likely to be located in areas with low EVI (Fig. 3b). For other mesopredators that rely on cover for hunting, survival might be higher in areas of dense vegetation; thus, future research should focus on how hunting behaviors and vegetation complexity interact to influence mesopredator survival.

In addition to habitat complexity affecting cheetah survival, we found that cheetah survival was also influenced by duration of exposure to top-down predation risk. Our results indicate that long-term risk of encountering a lion, rather than short-term risk of encountering a lion, influenced cheetah survival, with cubs having a lower probability of survival if there was a higher probability of encountering lions in their home range during the entire time when they were a cub. We likely did not observe effects of lion encounter risk on short-term cheetah survival because cheetahs have adapted behaviors to minimize short-term predation risk^25^. For example, cheetahs use the same general areas as apex predators such as lions and use fine-scale spatial partitioning to reduce the probability of lion encounters^10,26,27^. The use of spatial or temporal partitioning by mesopredators affected by apex predators has been found in a variety of other systems, such as red foxes (*Vulpes vulpes*) avoiding coyotes (*Canis latrans*) in North America^43^, and European badgers (*Meles meles*) avoiding wolves (*Canis lupus*) in Italy^11^.

Although fine-scale partitioning or other predator avoidance behaviors might be beneficial to reduce short-term risk for mesopredators, our results show that the long-term risk of co-occurring with an apex predator can negatively influence mesopredator survival. In the long-term, the risk of encountering lions could be associated with the direct effects of predation, with an increased probability of antagonistic encounters^44^. Long-term risk can also be associated with non-consumptive effects of predation related to reduced foraging^45,46^, or reduced parental care^47^. We could not explicitly investigate whether long-term exposure to predation risk reduced cub survival through direct or indirect effects. The majority of cub mortality in our study system is a result of predation^48^, but cubs also experience non-predation mortality such as starvation or injury^22,48^, and indirect effects of predation risk could have reduced cub body condition, which might increase susceptibility to predation. In the absence of direct predation, the long-term risk of predation has been found to cause changes in the morphology^49^, behavior^50,51^, physiology^52,53^, and demography^54,55^ of a variety of species. Our results build on the growing literature on long-term risk of predation to demonstrate how long-term predation risk can affect the demography of mesopredators.

Understanding spatial variation in survival can help inform wildlife conservation actions by focusing efforts on environmental factors that improve the survival of imperiled species. Specific for cheetahs in southern Africa, bush encroachment has caused the transition from open grasslands to closed habitats dominated by woody plants^56^. Bush encroachment can be caused by a number of factors including climate, fire, and herbivore distributions, but is predicted to increase based on future climate change models^57^. Based on our results, increased bush encroachment could be detrimental to cheetah populations that do not have adequate open areas for hunting. Thus, the persistence of this species in the southern portion of their range could be improved by prescribed burning or mechanical vegetation removal in order to maintain open habitats^58,59^. One limitation of our study was that we focused on only one cheetah population. Cheetahs experience variable conditions throughout their range, including differences in habitat composition, predator and prey communities, and conservation practices^60,61^. Therefore, further research is needed to better understand range-wide variability in spatial drivers of cheetah survival.

Our research shows how individual space use of mesopredators can scale-up and influence population-level processes, and illustrates the importance of understanding spatial drivers of survival on different temporal scales^62,63^. We propose that, in systems with apex predators and mesopredators, the survival of mesopredators in the short-term is driven by vegetative complexity likely associated with prey acquisition, whereas long-term survival depends on both top-down and bottom-up influences. Additionally, our results show that complex habitats might only be beneficial for mesopredators when they allow mesopredators to avoid apex predators, and effectively find and hunt prey, at the same time. Understanding how individual space use can influence population-level processes of mesopredators can offer insight into how communities with multiple predators are structured and can provide recommended conservation actions to ensure the future persistence of mesopredator species.

## Methods

### Study area

We studied cheetah survival in Mun-Ya-Wana Conservancy (Phinda Private Game Reserve), in northern KwaZulu-Natal, South Africa, from 2008–2018. The dominant vegetation type is broad-leaf woodland (42% of the conservancy), with open grasslands (31% of the conservancy), and semi-open wooded-grasslands (27% of the conservancy) interspersed throughout the reserve. The elevation of the Conservancy ranges from 4 to 201 m above sea level. The climate is subtropical with warm, dry winters (April–September) and hot, humid summers (October–March), with the majority of rain falling in the summer^64^. The Mun-Ya-Wana Conservancy is surrounded by electrified game fencing, and has grown in size as adjacent reserves have joined the Conservancy. From 2008–2017 the study area was 235 km^2^ in area, after which internal fences were removed and the Conservancy expanded to 285 km^2^. Cheetahs and lions were reintroduced into the reserve in 1992 and have been monitored since^48,65^. Throughout the study, lion densities ranged from 0.06 to 0.17 lions/km^2^ and cheetah densities ranged from 0.06 to 0.18 cheetahs/km^2^. Although both leopards (*Panthera pardus*) and spotted hyenas (*Crocuta crocuta*) occur in the study area, lions are the dominant apex predator^48,65^. Common prey species include impala (*Aepyceros melampus*), nyala (*Tragelaphus angasii*), zebra (*Equus quagga burchellii*), and wildebeest (*Connochaetes taurinus*).

### Carnivore monitoring

We monitored the cheetah and lion populations by subdividing the reserve into seven sections. Trained monitors usually drove the roads in each section at least once a week. In addition, monitors frequently followed-up on sightings reported by game rangers conducting game drives within the reserve. Cheetahs and lions can be individually recognized using their spot patterns, whisker spots, and scars, which allowed us to monitor the populations based on sightings alone^66^. We obtained an average of 40 ± 6 locations per individual cheetah during adult states and 27 ± 2 locations during cub states. When cheetahs or lions were observed, we recorded the location, behavior, and number of individuals present.

### Cheetah habitat use

We quantified coarse-scale cheetah habitat use by estimating lifetime home ranges for individual cheetahs. Although small-scale differences in habitat use might occur seasonally, cheetahs in our study area had stable home ranges across their lifetimes (Appendix S1). However, cheetahs will often shift home ranges when they become independent from their mothers, so for individuals that were included in the study as both cubs and adults, we estimated cub and adult home ranges separately. We estimated home ranges by calculating a utilization distribution (UD) using a fixed-kernel estimator and the plug-in method of bandwidth selection^67^. We only included cheetahs in our analyses with > 10 locations. For cubs died that before reaching the minimum number of locations, we used covariates associated with their mother’s home range, or the home range of their surviving littermates. For each cheetah’s home range, we extracted time-varying covariates of lion encounter risk, prey spatial density, and vegetation complexity (see below). To account for temporal differences in spatial drivers of survival, we extracted covariates within home ranges corresponding to each season, and also averaged covariates within home ranges across the lifetime of individual cheetahs. For cheetahs that were included in the analyses as both cubs and adults, we calculated separate “lifetime” covariate values for cub and adult periods separately. To identify the spatial scale most influential to survival, we extracted these covariates within the 50%, 75%, and 95% UD isopleths.

### Lion encounter risk

We estimated lion encounter risk by analyzing the spatial distribution of lions in each season^68,69^. We collected sightings data on the location of lion prides, rather than individual lions, from 2000–2019. For each pride of lions in a given season, we calculated a utilization distribution (UD) using a fixed-kernel estimator using the plug-in method of bandwidth selection^67^. To account for differences in pride size, we multiplied the UD for each pride by the average number of lions in that pride within a specific season^70^. To obtain a reserve-level measure of lion encounter risk, we added the individual pride UDs and rescaled the resulting values such that a value of 0 indicated no risk of encounter, and 1 indicated the highest risk of encounter.

### Prey spatial density

We estimated spatial variation in prey density in the reserve by collecting distance sampling data on impala, and nyala during the dry season (April–September), and the wet season (October–March) from 2010–2015^48^. We limited our prey analyses to these species because they comprised 82% of cheetah kills in the study area^65^. We estimated prey abundance using hierarchical distance sampling models with spatial covariates on both the abundance and detection processes, and used our top model to extrapolate prey abundance over our entire study period^48^. Our resulting prey density rasters depicted the average number of prey within 400 m^2^ cells for each season of each year.

### Vegetation complexity

We incorporated spatial variation in vegetation complexity into our cheetah survival models. Enhanced Vegetation Index (EVI) has been found to be correlated with vegetation structure in Africa, with open areas having low EVI values, and areas with dense vegetation having high EVI values^71^. In addition, EVI is sensitive to changes in rainfall. Thus, using EVI as an index for vegetation complexity also allowed us to incorporate changes in greenness, which could affect visibility. We obtained (EVI) data at a 250 m resolution (https://lpdaac.usgs.gov/data_access/data_pool) and calculated seasonal EVI values on a yearly basis by averaging EVI values across the entirety of a season.

### Cheetah spatially-explicit survival

We analyzed spatial drivers of cheetah monthly survival from February 2009 to March 2018. When a dead cheetah was discovered, we tried to determine the cause of death by checking the nearby area for tracks and scats, and examining the carcass. For each month, we recorded if each monitored cheetah was sighted or recovered dead as adults or cubs^48^. If a cheetah was removed from the reserve for management purposes, we censored that individual animal from analyses^48^. Because lion and cheetah density can vary greatly within a season, and because cubs can be born and become independent at any time during the year, we conducted our analysis on a monthly timescale to best reflect the conditions that might be driving survival^48^.

Because survival of cubs from the same litter might not independent, we first ran a Chi-square test of independent survival^72^ to test this assumption, with the null hypothesis being that survival of cubs is independent. To run this analysis, we randomly selected half (n = 19) of the monitored litters and ran a survival model (see below for information on model structure) without any individual covariates, to estimate monthly cub survival. We used the results of this model to estimate the expected number of living cubs at independence, which we defined as 16 months post-birth^48^. When then repeated this procedure 50 times and ran a Chi-square test on the observed vs. expected number of survived cubs. We found that fates of cubs within the same litter were independent (X^2^ = 34.3; p = 0.12), so for our subsequent survival models we treated each individual cheetah cub as an independent sample. Male cheetahs in the same coalition might also have non-independent fates, so we used the same Chi-square test of independent survival modeling framework with 50 replicates to test independence of males in coalitions (n = 11 coalitions). We found that that fates of males within the same coalition were independent (X^2^ = 6.5; p = 0.28), so for our subsequent analysis we treated each individual male cheetah as an independent sample.

### Model structure

Similar to previous research on cheetah survival in this system^48^, we analyzed cheetah survival using multi-state joint live-encounter dead-recovery models^73^ using the *rmark* R package^74^. This model made use of our frequent re-sightings and mortality data, and allowed for survival estimation based on individuals with unknown fates. Additionally, because juvenile cheetahs stay with their mothers for variable amounts of time^75^, we could not incorporate a regular age structure into our models. Thus, we used a multi-state approach to estimate survival for both cubs and adults simultaneously^48^. We specified the two model states as cub (juvenile cheetahs dependent on their mother) and adult (cheetahs that were independent from their mother) and did not incorporate immigration or emigration because our population was a closed population.

### Hypothesis testing

We previously determined that cheetah survival was best described using a structural model with resighting rate varying by year, and survival varying by season^48^. Therefore, we used the same structural model for these analyses to test for the effects of spatial covariates on cheetah survival. We used a two-stage approach to evaluate our hypotheses of interest. We first ran models to determine the spatial scale most influential to survival by running models with spatial covariates corresponding to the 50%, 75%, and 95% UD isopleths (Appendix S2). We considered spatial covariates on two temporal scales: short-term (seasonal), and long-term (spatial covariates within a home range averaged over an individual’s lifetime). The spatial scale associated with the best-fit model at both the short-term and long-term scales was retained and used for the hypothesis-testing portion of our analysis (Appendix S2).

To test our hypotheses of interest, we developed 11 a priori models that included covariates of average lion encounter risk, average prey density, and average EVI, as well as additive and multiplicative models with the same covariates. Similar to the first stage of our analysis, we considered spatial covariates both at the short-term (seasonal) and long-term (spatial covariates within a home range averaged over an individual’s lifetime) temporal scales. Because adults and cubs are known to have different survival rates^75^, we did not consider any models in which state was not included. We compared models separately for each temporal scale using Akaike’s Information Criterion corrected for sample size (AIC_c_;^76^), considered models within 2 ΔAIC_c_ of the top model to be competitive, and evaluated if covariates were informative by calculating 85% confidence intervals^77^.

## Supplementary information

Supplementary file1
