# Supplementary material for: Habitat complexity and lifetime predation risk influence mesopredator survival in a multi-predator system
Source: Sci Rep. 2020 Oct 20;10:17841. doi: 10.1038/s41598-020-73318-3 (PMC7575546; doi:10.1038/s41598-020-73318-3)
Supplement: Supplementary file 1 — Supplementary file1 [file 41598_2020_73318_MOESM1_ESM.docx]

Habitat complexity and lifetime predation risk influence mesopredator survival in a multi-predator system (Supplemental Information)

Laura C. Gigliotti^1^*, Rob Slotow^2^, Luke T. B. Hunter^2,3^, Julien Fattebert^2,4^, Craig Sholto-Douglas^5^, David S. Jachowski^1,2^

^1^ Department of Forestry and Environmental Conservation, Clemson University, 261 Lehotsky Hall, Clemson, SC, USA

^2^ Centre for Functional Biodiversity, School of Life Sciences, University of KwaZulu-Natal, Pietermaritzburg, South Africa

^3^ Wildlife Conservation Society, Bronx, NY, USA

^4^ Wyoming Cooperative Fish and Wildlife Research Unit, Department of Zoology and Physiology, University of Wyoming, Laramie, WY, USA

^5^ &Beyond Phinda Private Game Reserve, Hluhluwe, South Africa

*****Corresponding author: lcgigli@g.clemson.edu; Phone: 508-846-2184; Present Affiliation: Department of Environmental Science, Policy, and Management, University of California Berkeley, 3 Mulford Hall, Berkeley, CA 94720, USA

Appendix S1. Stability of cheetah home ranges

We analyzed lifetime variation in cheetah home ranges to determine if we could pool locations to estimate home ranges. For adult cheetahs with large sample sizes of locations (more than 30 locations in more than 2 years), we calculated lifetime home ranges, and yearly home ranges using a utilization distribution (UD) using a fixed-kernel estimator and the plug-in method of bandwidth selection ^1^. We quantified overlap between lifetime home ranges and yearly home ranges of individual cheetahs using Bhattacharyya’s affinity ^2^ (BA) because this metric has been found to be appropriate for quantifying similarity between utilization distributions ^3^. We calculated BA metrics for 50%, 75%, and 95% home range contours and considered a value of >0.60 to be indicative of high degrees of overlap. For all home range contours, cheetahs exhibited high overlap between yearly home ranges and lifetime home ranges (Table S1).

Appendix S2. Effects of spatial covariates at multiple scales on cheetah survival

To determine what spatial scale was most related to cheetah survival, we ran survival models using spatial covariates extracted from 50%, 75%, and 95% home range contours. We calculated home ranges using a utilization distribution (UD) using a fixed-kernel estimator and the plug-in method of bandwidth selection ^1^ and extracted lion density, prey density, and vegetation density covariates using the 50%, 75% and 95% home range contours. We analyzed cheetah survival using multi-state joint live-encounter dead-recovery models (see Methods for full details) using the spatial covariates at the three scales. We compared models using Akaike’s Information Criterion corrected for sample size ^4^ (AIC_c_), and considered models within 2 ΔAIC_c_ of the top model to be competitive

For all home range contours, short-term cheetah survival was most influenced by EVI (Table S2). When comparing all models simultaneously, the 50% home range contour models provided the best fit for the data, with the 75% home range contour models being competitive (Table S2).

Appendix S3. Effects of EVI on cheetah kill sites and locations where cheetahs were killed

We recorded the locations of cheetah kill sites (*n* = 664), and sites that cheetahs were killed by other predators (*n* = 32), from 1996 – 2019. We located kill sites by following hunting animals, or through opportunistic sightings of carcasses or feeding animals. When we could determine the species of predator and prey, we recorded the location using a handheld GPS.

We first investigated potential bias in our kill site data because of differing detection rates based on EVI. Because kill sites are usually first seen while driving on roads, we predicted that it would be easier to see kills sites further away from roads in areas of low EVI. For each recorded kill site, we calculated the distance to the nearest road, and extracted point-specific EVI values from the corresponding seasonal EVI raster. We investigated if there was a relationship between kill site EVI and distance to the nearest road using a Pearson’s product-moment correlation test using the *cor.test* function in Program R^5^. Based on this analysis there was no correlation between EVI and detection distance (r=0.0005, p=0.98), which shows that we were not more likely to detect kills sites at longer distances in areas of open vegetation. Therefore, we did not find any evidence to suggest that we needed to account for differences in kill site detection probability in relation to EVI.

For each season, we generated random points equal to the number of kill site locations or the number of locations where cheetahs were killed. We extracted EVI values from the corresponding season, at each used site and random site. We used logistic regression models to assess the effect of EVI on the probability of use using the *lm* function in Program R (Version 3.5.3) ^5^. We ran models separately for the cheetah kill site locations and the locations where cheetahs were killed. We evaluated if the EVI covariate was informative by calculating 85% confidence intervals, and considering it be informative if the confidence interval did not overlap zero ^6^. Locations of cheetah kill sites were negatively associated with EVI, with the highest probability of kills occurring in areas of low EVI. In contrast, there was no relationship between the locations where cheetahs were killed and EVI (β_EVI_ = 3.78; 85% C I= -1.47 to 9.27).

References

1. Gitzen, R. A., Millspaugh, J. J. & Kernohan, B. J. Bandwidth selection for fixed-kernel analysis of animal utilization distributions. *J. Wildl. Manage.* **70**, 1334–1344 (2006).

2. Bhattacharyya, A. On a measure of divergence between two statistical populations defined by their probability distributions. *Bull. Calcutta Mathe- matical Soc.* **35**, 99–109 (1943).

3. Fieberg, J. & Kochanny, C. O. Quantifying home-range overlap: the importance of the utilization distribution. *J. Wildl. Manage.* **69**, 1346–1359 (2005).

4. Burnham, K. P. & Anderson, D. R. *Model selection and multimodel inference: a practical information-theoretic approach*. *Ecological Modelling* **172**, (2002).

5. R Core Team. R: A language and environment for statistical computing. (2019).

6. Arnold, T. W. Uninformative parameters and model selection using Akaike’s Information Criterion. *J. Wildl. Manage.* **74**, 1175–1178 (2010).

Table S1. Lifetime and annual cheetah home range overlap based on Bhattacharyya’s affinity, Mun-Ya-Wana Conservancy, KwaZulu-Natal, South Africa, 2008 – 2018.

| Home range contour | Mean (± SE) Bhattacharyya’s affinity statistic |
| --- | --- |
| 50% | 0.76 ± 0.02 |
| 75% | 0.83 ± 0.01 |
| 95% | 0.89 ± 0.01 |

| Model (HR contour) | AICc | ΔAIC | Weight | k |
| --- | --- | --- | --- | --- |
| EVI (50%) | 3601.15 | 0 | 0.29 | 9 |
| EVI (75%) | 3602.17 | 1.04 | 0.17 | 9 |
| EVI (95%) | 3603.51 | 2.36 | 0.09 | 9 |
| Lion+EVI (50%) | 3603.51 | 2.36 | 0.09 | 11 |
| Lion*EVI (50%) | 3604.06 | 2.91 | 0.07 | 13 |
| Prey+EVI (50%) | 3604.60 | 3.45 | 0.05 | 11 |
| Prey+EVI (75%) | 3604.64 | 3.50 | 0.05 | 11 |
| Prey+EVI (95%) | 3605.36 | 4.21 | 0.04 | 11 |
| Lion+EVI (75%) | 3605.90 | 4.75 | 0.03 | 11 |
| Lion*EVI (95%) | 3606.28 | 5.13 | 0.02 | 13 |
| Prey*EVI (75%) | 3606.51 | 5.36 | 0.02 | 13 |
| Prey*EVI (95%) | 3606.86 | 5.69 | 0.02 | 13 |
| EVI+Lion+Prey (50%) | 3607.08 | 5.93 | 0.01 | 13 |
| Lion+EVI (95%) | 3607.18 | 6.03 | 0.01 | 11 |
| Lion*EVI (75%) | 3607.31 | 6.16 | 0.01 | 13 |
| Prey*EVI (50%) | 3607.31 | 6.16 | 0.01 | 13 |
| EVI+Lion+Prey (75%) | 3608.43 | 7.28 | 0.00 | 13 |
| EVI+Lion+Prey (95%) | 3608.96 | 7.81 | 0.00 | 13 |
| Lion*Prey (50%) | 3613.35 | 12.20 | 0.00 | 13 |
| Prey (50%) | 3617.96 | 16.81 | 0.00 | 9 |
| Prey (75%) | 3618.46 | 17.31 | 0.00 | 9 |
| Prey (95%) | 3618.49 | 17.34 | 0.00 | 9 |
| Lion (75%) | 3619.21 | 18.06 | 0.00 | 9 |
| Null (75%) | 3619.33 | 18.19 | 0.00 | 8 |
| Null (50%) | 3619.33 | 18.19 | 0.00 | 8 |
| Null (95%) | 3619.33 | 18.19 | 0.00 | 8 |
| Lion+Prey (95%) | 3619.97 | 18.82 | 0.00 | 11 |
| Lion (95%) | 3620.39 | 19.24 | 0.00 | 9 |
| Lion (50%) | 3620.62 | 19.47 | 0.00 | 9 |
| Lion+Prey (75%) | 3621.42 | 20.27 | 0.00 | 11 |
| Lion+Prey (50%) | 3621.51 | 20.36 | 0.00 | 11 |
| Lion*Prey (75%) | 3622.30 | 21.15 | 0.00 | 13 |
| Lion*Prey (95%) | 3622.84 | 21.69 | 0.00 | 11 |

Table S2. Model selection results to compare effects of covariates at multiple spatial scales using multi-state joint live-encounter dead-recovery spatial-explicit survival models for cheetahs with seasonal spatial covariates, Mun-Ya-Wana Conservancy, KwaZulu-Natal, South Africa, 2008 – 2018. States in the model include cubs (juveniles dependent on their mothers) and adults (non-juveniles). All models include effects of year on recovery rates and season on survival rates.
